# Prolonged Exposure to Elevated Iodine Levels in Drinking Water Is Associated With the Occurrence of Autoimmune Thyroid Disorders in Adults: Findings From a Case-Control Study Conducted in Shandong Province, China

**DOI:** 10.1155/jnme/1510663

**Published:** 2025-07-07

**Authors:** Yang Du, Peng Liu, Xu Huang, Fangang Meng, Lijun Fan, Weijia Li, Jinyin Yao, Xianglan Chen, Zhuowen Li, Ming Li, Chunpeng Lv, Wen Jiang, Wei Zhang, Dianjun Sun

**Affiliations:** ^1^Center for Endemic Disease Control, Chinese Center for Disease Control and Prevention, Harbin Medical University, Harbin, China; ^2^Key Lab of Etiology and Epidemiology, Education Bureau of Heilongjiang Province and Ministry of Health (23618504), Heilongjiang Provincial Key Lab of Trace Elements and Human Health, Harbin Medical University, Harbin, China; ^3^Department of Medical Records, Harbin Medical University Cancer Hospital, Harbin, China; ^4^Research Administration Department, Qilu Hospital (Qingdao), Cheeloo College of Medicine, Shandong University, Qingdao, China; ^5^Department of Public Health, Shandong Provincial Hospital Affiliated to Shandong First Medical University, Jinan, China; ^6^Department of Public Health, Guangdong Provincial People's Hospital Zhuhai Hospital, Zhuhai, China; ^7^Department of Endemic Diseases, Shandong Provincial Center for Disease Control and Prevention, Jinan, China

**Keywords:** AITD, case-control study, high iodine, risk factor, water iodine

## Abstract

**Background:** Excessive iodine intake is associated with an increased risk of thyroid autoimmunity. However, the relationship between prolonged exposure to iodine levels exceeding 100 μg/L in drinking water and the occurrence of autoimmune thyroid disorders (AITDs) remains uncertain. The objective of the present study was to assess whether elevated iodine levels exceeding 100 μg/L in drinking water are a risk factor for AITD.

**Methods:** We conducted a case–control study at a hospital, enrolling 668 adults. We measured serum thyroid peroxidase antibody (TPOAb), thyroglobulin antibody (TgAb), urinary iodine concentration (UIC), water iodine concentration (WIC), and serum cytokine concentrations—including interleukin-1β (IL-1β), interleukin-6 (IL-6), interferon-γ (IFN-γ), and tumor necrosis factor-α (TNF-α).

**Results:** The study demonstrated that individuals with water iodine levels > 100 μg/L had a significantly higher risk of developing AITD compared with those in the 10–100 μg/L group (OR = 2.076, *p* < 0.001). Furthermore, a family history of thyroid disorders (OR = 4.035, *p* < 0.001) and higher education levels (specifically college education compared to primary school; OR = 2.608, *p*=0.016) were associated with an increased risk of AITD. Conversely, regular consumption of freshwater fish was correlated with a lower risk of developing AITD (at least once per week vs. hardly eat, OR = 0.472, *p*=0.009; at least once per month vs. hardly eat, OR = 0.693, *p*=0.042). Additionally, IL-6 levels in the case group were significantly higher than those in the control group.

**Conclusions:** The case-control study demonstrated a significant association between the development of AITD and prolonged exposure to elevated iodine levels (above 100 μg/L) in water. AITD was found to be associated with several other factors. Risk factors for AITD include a family history of thyroid disorders and higher educational attainment. Additionally, the consumption of freshwater fish was identified as a protective factor. Identifying and understanding these significant risk and protective factors for AITD development are critical, and effective strategies should be developed and implemented for prevention and intervention targeting at-risk individuals.

## 1. Introduction

Iodine is an essential nutrient necessary for the biosynthesis of thyroid hormones, which are responsible for regulating growth, development, and metabolism [1]. An imbalance in iodine levels, either deficient or excessive, is known to contribute to thyroid disorders. Optimization of iodine intake in the population is critical to reducing the prevalence of thyroid diseases [2, 3]. Since the implementation of universal salt iodization (USI) in 1995, China has made significant strides in preventing iodine deficiency disorders (IDDs). To date, IDD has been effectively controlled at the national level and remains on the path toward elimination [4]. However, an increasing prevalence of iodine excess is being observed in China [5]. Excessive iodine intakes mainly result from the consumption of iodized salt, seafood that contains high levels of iodine, milk that is rich in iodine, and drinking water that is rich in iodine [6]. In China, there is a widespread distribution of areas with excessive iodine in drinking water. The National Drinking Water Iodine Survey reported high water iodine concentration (WIC) areas in nine provinces, including Tianjin, Hebei, Shandong, Jiangsu, Anhui, Henan, and Shanxi, among others, which posed a risk to approximately 30 million people [7]. Previous studies have examined the association between excessive iodine intake and autoimmune thyroid disorders (AITDs), demonstrating a potential link [8, 9]. However, previous studies relied on urinary iodine concentration (UIC) to assess the iodine nutritional status of the population. There is limited evidence linking individual water iodine to thyroid autoimmunity. According to the Chinese national health standard, areas with a median WIC above 100.0 μg/L are classified as regions with excessive water-borne iodine [10]. However, there is limited evidence supporting a direct link between WIC exceeding 100.0 μg/L and AITD.

This study aims to investigate the factors influencing the incidence of AITD through a case–control study. Specifically, it aims to examine the relationship between water iodine levels and AITD incidence, as well as explore the association between serum cytokine concentration and AITD.

## 2. Materials and Methods

### 2.1. Study Population

The case–control study was conducted from November 2019 to July 2021. Based on results from the 2017 national investigation of iodine levels in water sources, Shandong Province—with a particular focus on the city of Heze—was selected as a representative survey region to exemplify three distinct iodine concentration ranges in drinking water: < 10 μg/L, 10–100 μg/L, and > 100 μg/L. In these areas, the iodized salt supply was discontinued in 2009. This cessation can reflect the impact on residents' iodine nutrition through their consumption of drinking water. The case group comprised patients who were diagnosed with AITD in Heze municipal hospital for the first time based on laboratory tests and had resided in the selected regions for at least 5 years, aged between 18 and 60 years old. The control group consisted of individuals without thyroid disease and with normal thyroid function who were chosen from the same hospital. The control group was matched 1:1 with the case group based on age (±3 years) and sex.

Both the case group and the control group were excluded if they had any other diseases, disabilities, mental disorders, or autoimmune disorders that could potentially affect thyroid function. Additionally, informed consent was obtained from all subjects participating in the study.

### 2.2. Drinking Water and Urine Sample Collection and Determination

Participants provided water samples, which were collected in sterile 25 mL tubes and stored at 4°C until analysis. The iodine concentration in the drinking water was determined using the catalytic arsenic-cerium spectrophotometry method [11]. Nonfasting urine samples were collected from each participant between 7:00 and 10:00 a.m. Each urine sample contained more than 5 mL, was collected in a plastic tube, and stored at −20°C. The UIC was determined using the acid digestion method [12].

### 2.3. Questionnaire and Physical Examination

The questionnaire was administered through face-to-face interviews, encompassing demographic information, lifestyle factors, dietary habits, exposure to radiation, family history of thyroid diseases, source of drinking water, and drinking duration. Weight and height were measured in kilograms and centimeters, respectively. The body mass index (BMI) was calculated by dividing the weight (in kilograms) by the square of the height (in meters).

### 2.4. Blood Sample Collection and Analysis

Fasting blood samples were collected from the antecubital veins of the adult participants in the morning and stored in glass tubes. Serum samples were prepared by centrifuging them at 3000 rpm for 10 min after standing for 1 h and subsequently freezing them at −80°C. The frozen serum samples were subsequently transported to Harbin Medical University in Heilongjiang Province, where they were stored in a −80°C freezer until analysis. The Fourth Affiliated Hospital of Harbin Medical University measured serum concentrations of serum thyroid peroxidase antibody (TPOAb) and thyroglobulin antibody (TgAb) using chemiluminescent immunoassays (Roche Diagnostic GmbH, Germany). The reference ranges for TGAb and TPOAb were 0–115.0 IU/mL and 0–34.0 IU/mL, respectively.

### 2.5. Diagnostic Criteria for AITD

AITD: TPOAb and/or TgAb positive.

### 2.6. Analysis of Serum Cytokines

Four cytokines—namely, interleukin-1β (IL-1β), interleukin-6 (IL-6), interferon-γ (IFN-γ), and tumor necrosis factor-α (TNF-α)—were evaluated using the MILLIPLEX MAP Human Cytokine/Chemokine/Growth Factor Panel A (HCYTA-60K-PX38, Merck KGaA, Darmstadt, Germany) according to the manufacturer's guidelines. The data were analyzed using Belysa software (Merck KGaA, Darmstadt, Germany) on the MAGPIX instrument.

### 2.7. Statistical Analysis

The questionnaire data and the data from general physical examinations were entered into the Epi Info 7.2 software to establish a comprehensive database. The data were analyzed via SPSS 19.0 statistical software, while conditional logistic regression and mediation effect analysis were conducted using R 4.4.2. Descriptive statistics for normally distributed data are typically presented as the mean ± standard deviation, and a comparison between two groups is typically conducted using the independent samples *t*-test. For non-normal data, median and interquartile ranges were used, and a comparison between the two groups was conducted using the Mann–Whitney *U* test. The distribution of data was assessed using the chi-square test. Statistical significance is defined as *p* < 0.05.

## 3. Results

### 3.1. Description of the Participants

The demographic characteristics of the control and case groups are summarized in Table 1. This study included a total of 334 pairs of AITD patients and healthy controls, comprising 66 males and 602 females, resulting in a male/female ratio of 1:9.1. Statistically significant differences were observed in both education level distribution (*p* < 0.001) and family history of thyroid disorders (*p* < 0.001). The analysis indicated no statistically significant difference in medical radiation exposure between the case group and the control group. Nonetheless, a statistically significant difference was found in the duration of mobile phone and computer usage (*p*=0.022).

### 3.2. Iodine Levels in Water and Urine in the Control Group and the Case Group

The median levels of water iodine and urine iodine in the control group were 98.7 μg/L and 288.5 μg/L, while in the case group, they were 160.0 μg/L and 324.5 μg/L, respectively. Nonparametric tests revealed a statistically significant difference in the water iodine level between the case and control groups (*p*=0.001). Statistical differences were observed in the levels of urine iodine (*p*=0.042). Chi-square tests revealed significant disparities in the proportion of water iodine levels between the case and control groups (*p* < 0.001) (Table 2).

### 3.3. The Dietary Habits of the Control Group and the Case Group

Table 3 presents the dietary patterns exhibited by both the control and case groups. A remarkable disparity (*p*=0.009) surfaced in the frequency at which freshwater fish was consumed when scrutinizing the case group in comparison to the control group.

### 3.4. Univariate Logistic Regression Analysis of Factors Influencing AITD

The univariate logistic regression analysis took into account possible factors related to the onset of AITD, including WIC, education level, family history of thyroid disorders, duration of mobile phone and computer usage, and frequency of freshwater fish consumption (Table 4). The results indicated that risk factors for AITD included WIC > 100 μg/L, a higher education degree, a family history of thyroid disorders, and the use of a mobile phone and computer for at least 2 h per day. The consumption of freshwater fish served as a protective factor against AITD.

### 3.5. Multiple Logistic Analysis of Factors Influencing AITD

To further analyze the factors associated with AITD, a multiple logistic regression analysis was performed, including WIC, education level, family history of thyroid disorders, frequency of freshwater fish consumption, and duration of mobile phone and computer use (Table 5). The results of the multiple logistic regression suggested that individuals from the water iodine exceeding 100 μg/L group are more likely to suffer from AITD compared to those in the 10–100 μg/L group (OR = 2.076, *p* < 0.001). Moreover, having a family history of thyroid disorders (OR = 4.035, *p* < 0.001) and a higher level of education, specifically college versus primary school (OR = 2.608, *p*=0.016), were risk factors for developing AITD. However, consuming freshwater fish was associated with a reduced risk of AITD (at least once per week vs. hardly eat, OR = 0.472, *p*=0.009; at least once per month vs. hardly eat, OR = 0.693, *p*=0.042).

### 3.6. Association Between Family History and AITD Prevalence in the WIC > 100 μg/L Subgroup

No statistically significant difference was observed in AITD prevalence between participants with a positive family history (75.0%) and those with a negative family history (57.0%) (*p*=0.063) (Table 6).

### 3.7. The Levels of Cytokines in the Control Group and Case Group

When comparing the levels of cytokines between the case and control groups, the concentrations of IL-6 in the case group were significantly higher than those in the control group (*p*=0.001). There was no significant difference in IL-1β, TNF-α, and IFN-γ levels between the control group and the case group (Table 7).

### 3.8. Mediation Analysis of WIC and AITD: The Role of IL-6

Mediation analysis employing a quasi-Bayesian approach with 1000 Monte Carlo simulations disclosed that IL-6 did not significantly mediate the association between WIC and the incidence of AITD after accounting for age, gender, and BMI [average causal mediation effect (ACME) = 0.0002, 95% CI: −0.009–0.01, *p*=0.994; proportion mediated = 0.014%, 95% CI: −31.6%–23%]. The analysis evidenced a significant direct effect of WIC on the risk of AITD [average direct effect (ADE) = 0.057, 95% CI: 0.015–0.10, *p*=0.006], accounting for more than 99% of the total effect (total effect = 0.057, 95% CI: 0.014–0.10, *p*=0.012). These findings suggest that the observed association between chronic iodine exposure and AITD predominantly occurs via biological pathways independent of IL-6-mediated inflammation.

## 4. Discussion

Iodine is an indispensable micronutrient for human beings and plays a vital role in thyroid hormone synthesis. Iodine predominantly exists in water-soluble compounds in the natural environment. The present study identified a significant disparity in the median WIC between the case group and the control group. The case group exhibited a markedly elevated median WIC in comparison to the control group. Correspondingly, a significant difference was observed in urinary iodine levels between the case group and the control group. Additionally, the presence of high levels of water iodine, exceeding 100 μg/L, was identified as a potential risk factor for AITD. Similar observations were also made in China. In a study conducted in China, it was observed that adults residing in areas with excessive iodine intake had a higher prevalence of TPOAb and/or TgAb positivity compared to their counterparts living in areas with adequate iodine intake [3].

The results of this study suggest that having a family history of thyroid disorders and attaining a university education are independently associated with an increased risk of AITD. Consistent with these findings, prior studies have shown the family aggregation of AITD. Siblings of patients were found to have a disease risk ratio (RR) of approximately 17 and a 50% higher positivity rate of thyroid autoantibodies [13]. Furthermore, a study involving twins estimated a heritability rate of approximately 79% for Graves' disease (GD) and around 70% for the presence of thyroid autoantibodies [14], which further supports this hypothesis. The present study identified a higher risk of AITD development among individuals with a university education level, indicating a potential association with elevated work-related stress levels. This finding aligns with previous reports that documented a greater occurrence of stressful events during the year preceding GD diagnosis when compared to a healthy control group [15]. However, if one lives in an area with high water iodine, his family is probably also exposed to high water iodine, so the prevalence of thyroid disease among his family members may also be higher. A comparison of the prevalence rates in the subgroup with WIC > 100 μg/L between those with family history and those without was carried out in this study. The results reveal that within the subgroup exhibiting WIC levels exceeding 100 μg/L, the prevalence of AITD among individuals with a family history of thyroid disorders does not demonstrate a statistically significant difference from that of those without such a history. This result may be due to the limited sample size in the family-history-positive subgroup, reducing statistical power and potentially obscuring a true association. Future studies should assess family iodine exposure to disentangle genetic and environmental effects. Additionally, the study revealed that regular consumption of freshwater fish was associated with a reduced risk of AITD. Fish is widely recognized as the primary dietary source of omega-3 polyunsaturated fatty acids (PUFAs). Multiple studies indicate that the consumption of PUFAs, particularly omega-3 fatty acids, appears to exert protective effects on the pathogenesis of autoimmune thyroiditis [16–18]. The association between smoking or alcohol consumption and AITD remains controversial. Smoking and alcohol have significant effects, both potentially protective and exacerbating the disease, although the precise nature of these effects is not known [19]. This study did not find a significant association between smoking or alcohol consumption and AITD.

Cytokines play pivotal roles in immune regulation and maintenance of immunological homeostasis, with particular relevance to thyroid pathologies through their direct actions on thyroid follicular cells [20, 21]. Previous studies have indicated that IL-6 is associated with the apoptosis of thyroid follicular cells by activating the Janus kinase-signal transducer and activator of transcription (JAK–STAT) pathway and may contribute to Th17/Treg immune disequilibrium [22]. Our findings demonstrate significantly elevated serum IL-6 concentrations in AITD patients compared to those in healthy controls, consistent with previous reports of iodine-induced IL-6 upregulation in thyroid cells [23]. However, mediation analysis revealed no significant role of IL-6 in linking water iodine to AITD, suggesting that IL-6 elevation is more likely a consequence of thyroid tissue damage (e.g., via DNA damage [23]) than a causal intermediary. This interpretation aligns with experimental evidence that iodine excess directly induces oxidative stress and NF-κB activation in thyroid cells [24, 25], which may promote immune activation through pathways independent of IL-6. Thus, while IL-6 levels are elevated in AITD, their role appears secondary to iodine's direct effects on thyroid tissue. These findings reinforce the concept that excessive iodine intake contributes to AITD development through complex immunomodulatory pathways, with IL-6 serving as a biomarker of disease activity rather than a mediator of iodine's effects. Further investigation is required to elucidate whether iodine's direct immunogenicity or interactions with unmeasured inflammatory mediators (e.g., NF-κB) drive this process.

The present study was strengthened by selecting survey villages that were provided with noniodized table salt, instead of iodized salt, to mitigate its impact. In these areas, it was found that water constituted the primary source of high iodine intake, in contrast to iodized salt. Nevertheless, the study had certain limitations. The iodine content of the food was not assessed in the study. Moreover, focusing primarily on female participants in the study may introduce potential bias in reporting their lifestyle habits. Additionally, this study did not examine the impact of certain environmental factors, such as selenium (Se), which has the potential to influence thyroid autoimmunity [26].

## 5. Conclusions

We demonstrated that excess iodine is a risk factor for AITD, particularly in areas where water iodine levels exceed 100 μg/L. Additionally, other factors, such as a family history of thyroid disorders, higher educational attainment, and freshwater fish consumption, were found to be associated with AITD. Therefore, identifying significant risk and protective factors for AITD—especially modifiable ones—is essential for developing targeted prevention strategies. Currently, IDDs have been successfully controlled and are being sustainably eliminated in China. Nonetheless, there is an excess of iodine in areas with high water iodine levels. Merely supplying noniodized salt is insufficient to shield residents from the detrimental effects of excess iodine in areas where the median water iodine content exceeds 100.0 μg/L. It is imperative to implement effective measures in these areas, such as shifting to a safe water source or reducing the iodine content in drinking water. Given that excess iodine status can elevate the risk of AITD, diligent monitoring of water iodine is crucial to prevent iodine-related harm and mitigate its adverse impact.

## Figures and Tables

**Table 1:** Demographic characteristics of the participants.

Characteristics		Control group (*n* = 334)	Case group (*n* = 334)	t or *χ*^2^	*p* value
Gender (*n*, %)	Male	33 (9.9)	33 (9.9)		
	Female	301 (90.1)	301 (90.1)		

Age (years, mean ± SD)		43.5 ± 10.4	44.8 ± 10.5	1.637	0.102

BMI (kg/m^2^, mean ± SD)		25.1 ± 3.8	25.1 ± 4.1	0.063	0.95

Education level (*n*, %)	Primary school	157 (47.0)	158 (47.3)	18.848	**< 0.001**
	Junior high school	130 (38.9)	100 (29.9)		
	Senior high school	36 (10.8)	38 (11.4)		
	College	11 (3.3)	38 (11.4)		

Smoking (*n*, %)	No	304 (91.0)	315 (94.3)	2.665	0.103
	Yes	30 (9.0)	19 (5.7)		

Drinking (*n*, %)	No	295 (88.3)	288 (86.2)	0.661	0.416
	Yes	39 (11.7)	46 (13.8)		

Family history of thyroid disorders (*n*, %)	No	322 (96.4)	290 (86.8)	19.959	**< 0.001**
	Yes	12 (3.6)	44 (13.2)		

Radiation from medical sources (*n*, %)	No	174 (52.1)	191 (57.2)	1.746	0.186
	Yes	160 (47.9)	143 (42.8)		

Duration of mobile phone and computer usage (*n*, %)	< 2 h/day	217 (65.0)	188 (56.3)	5.274	**0.022**
	≥ 2 h/day	117 (35.0)	146 (43.7)		

*Note:* Bold values represent *p* < 0.05.

**Table 2:** The water iodine and urinary iodine in the control group and case group.

Group		Control group (*n* = 334)	Case group (*n* = 334)	*p* value
Water iodine (μg/L)	Median (interquartile)	98.7 (18.0, 177.0)	160.0 (36.4, 243.1)	0.001^a^
	< 10 μg/L	68 (20.4)	43 (12.9)	<0.001^**b**^
	10–100 μg/L	114 (34.1)	78 (23.4)	
	> 100 μg/L	152 (45.5)	213 (63.8)	
Urinary iodine (μg/L)	Median (interquartile)	288.5 (189.0, 423.3)	324.5 (198.8, 488.3)	0.042^a^

*Note:* Bold values represent *p* < 0.05.

^a^ *p* values were calculated using the Mann–Whitney *U* test.

^b^ *p* value was calculated using the chi-square test.

**Table 3:** Dietary patterns in the case group and the control group.

Category		Control group (*n* = 334)	Case group (*n* = 334)	*χ* ^2^	*p* value
Frequency of consuming kelp (*n*, %)	At least once per week	27 (8.1)	19 (5.7)	2.679	0.262
	At least once per month	74 (22.2)	88 (26.3)		
	Hardly eat	233 (69.8)	227 (68.0)		

Frequency of consuming seaweed (*n*, %)	At least once per week	38 (11.4)	28 (8.4)	1.749	0.417
	At least once per month	87 (26.0)	87 (26.0)		
	Hardly eat	209 (62.6)	219 (65.6)		

Frequency of consuming freshwater fish (*n*, %)	At least once per week	53 (15.9)	32 (9.6)	9.347	**0.009**
	At least once per month	172 (51.5)	162 (48.5)		
	Hardly eat	109 (32.6)	140 (41.9)		

Frequency of consuming eggs (*n*, %)	At least once per day	171 (51.2)	147 (44.0)	5.361	0.147
	At least once per week	115 (34.4)	133 (39.8)		
	At least once per month	21 (6.3)	31 (9.3)		
	Hardly eat	27 (8.1)	23 (6.9)		

Frequency of consuming milk (*n*, %)	At least once per day	17 (5.1)	17 (5.1)	3.834	0.28
	At least once per week	45 (13.5)	51 (15.3)		
	At least once per month	38 (11.4)	53 (15.9)		
	Hardly eat	234 (70.1)	213 (63.8)		

Frequency of consuming seafood (*n*, %)	At least once per week	21 (6.3)	15 (4.5)	1.289	0.525
	At least once per month	82 (24.6)	89 (26.6)		
	Hardly eat	231 (69.2)	230 (68.9)		

Taste (*n*, %)	Salty taste	123 (36.8)	134 (40.1)	2.962	0.227
	Slightly bland	107 (32.0)	116 (34.7)		
	Moderate	104 (31.1)	84 (25.1)		

*Note:* The bold value represents *p* < 0.05.

**Table 4:** Univariate logistic regression analysis on factors affecting AITD.

Variable		β	*p* value	OR	95% CI
Water iodine	< 10 μg/L vs.10–100 μg/L	−0.079	0.747	0.924	0.573–1.491
	> 100 μg/L vs.10–100 μg/L	0.717	**< 0.001**	2.048	1.436–2.922

Family history of thyroid disorders	Yes vs. no	1.404	**< 0.001**	4.071	2.109–7.859

Education level	Junior high school vs. primary school	−0.269	0.123	0.764	0.543–1.076
	Senior high school vs. primary school	0.048	0.854	1.049	0.632–1.741
	College vs. primary school	1.233	**0.001**	3.433	1.694–6.957

Duration of mobile phone or computer usage	≥ 2 h/d vs. < 2 h/d	0.365	**0.022**	1.44	1.054–1.968

Frequency of consuming freshwater fish	At least once per week vs. hardly eat	−0.755	**0.003**	0.47	0.284–0.779
	At least once per month vs. hardly eat	−0.31	0.065	0.733	0.527–1.02

*Note:* Bold values represent *p* < 0.05.

**Table 5:** Multiple logistic regression analysis of factors influencing AITD.

Variable		β	*p* value	OR	95% CI
Water iodine	< 10 μg/L vs. 10–100 μg/L	0.02	0.94	1.02	0.605–1.713
	> 100 μg/L vs. 10–100 μg/L	0.73	**< 0.001**	2.076	1.418–3.057

Family history of thyroid disorders	Yes vs. no	1.395	**< 0.001**	4.035	2.08–8.379

Education level	Junior high school vs. primary school	−0.33	0.092	0.719	0.489–1.054
	Senior high school vs. primary school	−0.023	0.935	0.977	0.558–1.711
	College vs. primary school	0.958	**0.016**	2.608	1.225–5.909

Duration of mobile phone or computer usage	≥ 2 h/day vs. < 2 h/day	0.302	0.102	1.352	0.942–1.945

Frequency of consuming freshwater fish	At least once per week vs. hardly eat	−0.751	**0.009**	0.472	0.268–0.826
	At least once per month vs. hardly eaten	−0.366	**0.042**	0.693	0.487–0.987

*Note:* Bold values represent *p* < 0.05.

**Table 6:** Comparison of AITD prevalence in the WIC > 100 μg/L subgroup stratified by family history.

AITD status	Family history (+)	Family history (−)	*p* value
Affected (yes) *n* (%)	21 (75.0)	192 (57.0)	0.063
Unaffected (no) *n* (%)	7 (25.0)	145 (43.0)	

**Table 7:** The levels of cytokines in the control group and case group.

	Control group Median (interquartile)	Case group Median (interquartile)	*p* value
IL-1β (pg/mL)	7.2 (3.9, 15.7)	8.4 (3.5, 37.6)	0.302
IL-6 (pg/mL)	2.3 (1.1, 5.0)	3.7 (1.3, 10.3)	**0.001**
IFN-γ (pg/mL)	1.6 (0.8, 2.8)	2.0 (0.7, 5.7)	0.186
TNF-α (pg/mL)	14.8 (8.3, 20.5)	15.7 (9.1, 25.2)	0.345

*Note:* The bold value represents *p* < 0.05.

## Data Availability

The data that support the findings of this study are available on request from the corresponding authors. The data are not publicly available due to privacy or ethical restrictions.
